# History of the Nerve Block

**DOI:** 10.52965/001c.125260

**Published:** 2024-11-11

**Authors:** Daniel Go, Murdoc Gould, Latha Ganti

**Affiliations:** 1 Lake Highland Preparatory Schooi; 2 Birkbeck, University of London; 3 Orlando College of Osteopathic Medicine; 4 Brown University

**Keywords:** nerve block, regional anesthesia, history of medicine

## Abstract

Nerve blocks hold an important place in medicine. They are used to help with surgeries, allowing a painless surgical procedure without requiring the patient to be on full anesthesia. They can also provide a faster recovery period and apply to almost any body part. This paper summarizes how the nerve block became a procedure, the history of the drugs used and developed for it, and how machinery has allowed nerve blocks to progress to the point they are at today.

## Introduction

Nerve blocks, also known as neural blockades, are used as regional anesthesia to limit pain by injecting medicines into specific nerves to restrict their activity.^1^ Blocks can be performed in various locations, including around the elbows, knees, and ankles. They all share the same goal: blocking the local nerves in the area of interest to prevent pain from being registered by the brain. This can allow patients to recover quicker than normal since they are not under the effects of heavy anesthesia, need less medication at home, and have better pain control.^2^ This procedure can be used in several surgeries and procedures, the most notable being a femoral nerve block for an anterior cruciate ligament (ACL) surgery and epidural blocks during labor and delivery upon request.^3^ As this practice is commonly used for a variety of surgeries, it is important to understand the history and development of anesthetic blocking, as well as what other changes may be made later on.

**Figure 1.**
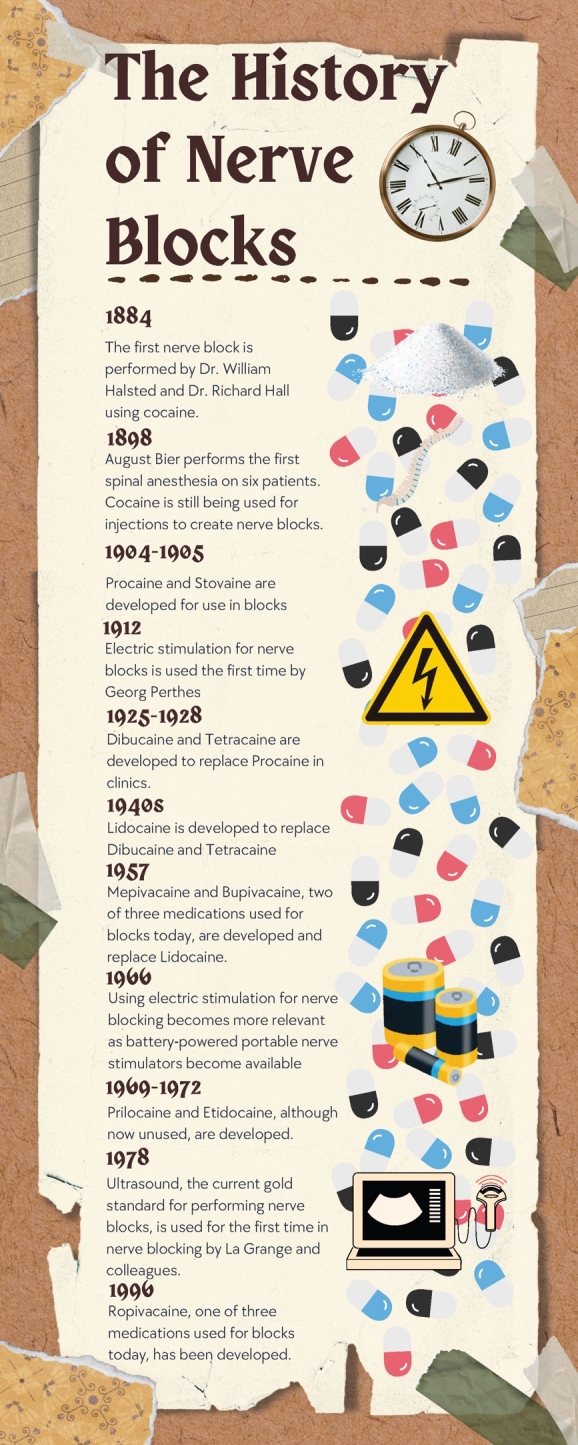
Infographic summarizing the history of nerve blocks Created by Daniel Go in Canva.com

## Procedure

Cocaine has been known to have anesthetic properties for a long time, which was first discovered in 1653 when Spanish Jesuit Bernabé Cobo found that chewing on the coca plant leaves would alleviate toothaches and pain.^4^ It wasn’t until 1884, however, that the anesthetic properties of cocaine were first surgically utilized by surgeon Dr. William S. Halsted to perform the first nerve block. Dr. Halsted directly injected cocaine into the anterior superior dental nerve and inferior alveolar nerve before surgeries, successfully blocking the nerve and limiting pain during and after surgery. His aide, Dr. Richard John Hall, assisted Dr. Halsted when first blocking a patient.^4,5^ The popularity of cocaine as an anesthetic, which was boosted by other doctors earlier in the year, is what ultimately led to the creation of nerve blocking.

During the late nineteenth century, many doctors were starting to utilize cocaine as an anesthetic, and many doctors gave talks on cocaine’s use in the medical industry. Carl Koller testified to this fact during a presentation to the Congress of Ophthalmology in Heidelberg about using cocaine as an anesthetic in 1884. Dr. Henry D. Noyes, a doctor present at this presentation, also had a letter published in the New York Medical Records about cocaine use before this presentation.^6^ As a result of all of these findings, Dr. Halsted and Dr. Hall started clinical trials officially researching the use of cocaine to create a regional anesthetic, injecting four percent solutions of cocaine into nerves to limit pain.^7^ This was the first development of a nerve block, and it did not take long for the technique to become widely used and develop further.

The nerve block continued to progress in 1898 when August Bier used cocaine to perform spinal anesthesia on his two children, as well as four other patients. In fact, because of his success with spinal anesthesia, the block Dr. Bier created is now called a Bier block.

Anesthesia in the form of blocks also happened to begin to raise awareness for children going through surgery, as it was a belief during this time that children actually could not feel pain.^8^ From 1904-1905, spinal anesthesia started to use a variety of other medications, with procaine and stovaine being used rather than cocaine. During the early development of nerve blocks in the late 1800s and early 1900s, performing the procedure was a job that was assigned to surgeons rather than anesthetists.^7^ This only really changed when anesthesia started becoming a specialty from 1930-1955, separating fully from surgery as a practice. As of 1910, surgeons performed nerve blocks. Surgeons, however, were the main reason that nerve blocks became widely accepted. This is because many surgeons explained blocks as a way to make patients extremely comfortable during surgery - to the point that they could eat cake during it.^9^ After the block became well-known and widely used, there was not as much development in the technique of administering the block. Different kinds of blocks were developed, leading to peripheral nerve blocks and regional nerve blocks. Peripheral nerve blocks are much more specific than regional nerve blocks. For example, regional nerve blocks, including spinal blocks, can numb as far as your entire waist down, while peripheral nerve blocks are much more specific in the area they affect, allowing anesthetists to numb smaller areas, such as the patient’s foot.

Even with these developments in nerve block categories, the main changes in nerve blocks from this point on were with the medications used and machinery that made the process easier than before.

## Drugs Used

Cocaine, the first drug used in blocking, had limited use due to how addictive it was, leading to the development of procaine in 1904 and the use of it in 1905. Procaine provided the same benefits as cocaine without causing addiction, which led to it being the most popular substitute for cocaine during this period. The main issue with procaine that led to the development of other medicines was that it didn’t last nearly as long as cocaine. On top of that, the use of procaine had a chance of creating local allergic reactions, which would hinder surgery and recovery. These issues led to the creation of dibucaine in 1925 and tetracaine in 1928. Although these medicines did fix the initial issue with the length of time the patient was under the medication’s effect, it also suffered from being allergenic. This continued to be the trend with medications used in nerve blocks until lidocaine was developed during the mid-1940s, which was revolutionary as it was the first that had a long duration of effect and had an incredibly low chance of causing an allergic reaction. Unfortunately, there were still several issues that lidocaine caused, with fever, fast heartbeat, and difficulty breathing being a few of the issues.^10^ To combat side effects, more medications were developed, such as mepivacaine (created in 1957), bupivacaine (created in 1957), prilocaine (created in 1969), and etidocaine (created in 1972). The only medicines from these four that are still used today are mepivacaine and bupivacaine. Although these medications do well in providing a nerve block, there are still issues with them, including reduced motor function and heart toxicity, which limits the amount that can be given to patients. As a result of these issues, ropivacaine was developed in 1996 and is now more commonly used since it causes less motor blockade and decreases the potential for cardiac toxicity. The three main medicines used today are bupivacaine, ropivacaine, and mepivacaine.^9^ The other evolutions in nerve blocks mainly come from the evolution of the machinery that is used alongside it, allowing nerve blocks to be done more easily and accurately.

## Technique

Initially, it was much harder to know if a nerve block was done correctly. A lot of anesthesiologists had to rely on patient feedback to know if they had inserted the needle in the correct place, with the most traditional techniques relying on feeling subsequent pops and clicks as the anesthesiologists moved their needle, which were very subjective and relatively difficult to gauge.^11^ This technique started evolving to make blocking easier and easier over time, starting with the use of nerve stimulation. The first example of nerve stimulation was in 1780 when Luigi Galvani found that applying an electric current to a frog’s muscles would cause twitching because of the muscle contracting repeatedly, a technique that started to be used in the 1900s to help with administering blocks, with the first recorded use of electrical stimulation for nerve blocks being in 1912 by Georg Perthes in Germany. Using this stimulation made it easier to identify the nerve and administer medication in the correct location, but it did not become a common clinical practice until 1966 when battery-powered portable nerve stimulators became available. This progress increased the chance of a successful nerve block and reduced the amount of medication needed for a block, which made it increasingly popular in most clinics. The development of this technique resulted in the worldwide growth of interest in nerve blocking as a technique, leading to more clinics utilizing it as a way to limit their patient’s pain and medication dose. On top of this, using electrical stimulation made it less likely that the patient would get injured due to the needle or injection, along with a decreased amount of nerve trauma.^11^ The current gold standard is ultrasound, which can be used to actively see the nerve, and was first used in 1978 by La Grange and his colleagues.^12^ Using ultrasound as a technique allows the nerve to be identified before any medication is injected, which minimizes the amount of medication that is used on the patient. Along with increasing the benefits that would normally be gained from using nerve stimulation (being more accurate with less of a chance of injury and less medication), using ultrasound also decreased block set-up time and increased the amount of time a block was active. Unfortunately, the use of ultrasound is much more limited than with other techniques, as machinery isn’t always available, and the use of machinery requires proper training. Complications with injections include infection, bleeding, and even death due to local anesthetic systemic toxicity, a rare case that can be caused by local anesthesia.^13^

As of right now, the nerve block procedure uses a syringe that is used to inject medicine around the area of a nerve. The medications that are currently used include alpha-2 agonists, dexamethasone, and midazolam, but many places also include anti-inflammatory medication to ensure fast recovery.^13^ The procedure has remained the same throughout the years, with the current technique still being done for fast recovery and less heavy anesthetic.^13^

There are, however, a number of new advancements that have been made in recent years. One of these includes the use of a new drug: liposomal bupivacaine. This drug is used in nerve blocks for the purpose of providing better and longer-lasting pain control, which it does by using multi-vesicular liposomes that contain bupivacaine.^14^ As a result of this medication, pain relief lasts for 72-96 hours, which can help in aiding long operations and improve the safety of operations.^14^ On top of this, there are several new blocking techniques that have been tested for pain prevention and effectiveness, which suggests that there will be a major advancement regarding blocking shortly.^15^ One of the blocks that is being tested is the parenteral block in the hip, which provides a hip block that takes effect after 15 minutes of being done.^16^ Having a slow-acting block could help in making sure that the patient is comfortable and that there is not an allergic reaction in the patient before continuing.

## Conclusion

Starting from inserting a cocaine-filled syringe around someone’s nerve ending to have a chance at blocking pain and evolving into a fully developed technique using ultrasound, electricity, and developed medications that can be applied in numerous surgeries, the nerve block remains one of the most important procedures for anesthesiologists to know and perform. Over the next few years, the procedure may see even more evolution, whether it will be performed properly in any place without the need for equipment or if it means new drugs will be developed in place of the current ones. Currently, there are some issues with using ultrasound, as there can be cases where the machine overloads, presenting opportunities to find better methods for identifying nerves for blocking purposes. No matter what developments are made, it remains clear that this technique will remain relevant in the medical industry in the foreseeable future.
